# Shape Memory Performance and Microstructural Evolution in PLA/PEG Blends: Role of Plasticizer Content and Molecular Weight

**DOI:** 10.3390/polym17020225

**Published:** 2025-01-17

**Authors:** Jiradet Sringam, Todsapol Kajornprai, Tatiya Trongsatitkul, Nitinat Suppakarn

**Affiliations:** 1 School of Polymer Engineering, Institute of Engineering, Suranaree University of Technology, Nakhon Ratchasima 30000, Thailand; jiradet@g.sut.ac.th (J.S.); kajornprai.t@gmail.com (T.K.); tatiya@sut.ac.th (T.T.); 2 Research Center for Biocomposite Materials for Medical Industry and Agricultural and Food Industry, Nakhon Ratchasima 30000, Thailand

**Keywords:** PLA, PLA/PEG blend, shape memory polymer, synchrotron WAXS

## Abstract

Poly(lactic acid) (PLA) exhibits excellent shape memory properties but suffers from brittleness and a high glass transition temperature (T_g_), limiting its utility in flexible and durable applications. This study explored the modification of PLA properties through the incorporation of poly(ethylene glycol) (PEG), varying in both content (5–20 wt%) and molecular weight (4000–12,000 g/mol), to enhance its suitability for specific applications, such as medical splints. The PLA/PEG blend, containing 15 wt% PEG and with a molecular weight of 12,000 g/mol, exhibited superior shape fixity (99.27%) and recovery (95.77%) in shape memory tests conducted at a programming temperature (T_p_) of 45 °C and a recovery temperature (T_r_) of 60 °C. Differential scanning calorimetry (DSC) analysis provided insights into the thermal mechanisms driving shape memory behavior of the PLA/PEG blend. The addition of PEG to the PLA blend resulted in a reduction in T_g_ and an increase in crystallinity, thereby facilitating enhanced chain mobility and structural reorganization. These thermal changes enhanced the shape fixity and recovery of the PLA/PEG blend. Synchrotron wide-angle X-ray scattering (WAXS) was further employed to elucidate the microstructural evolution of PLA/PEG blends during the shape memory process. Upon stretching, the PLA/PEG chains aligned predominantly along the tensile direction, reflecting strain-induced orientation. During recovery, the PLA/PEG chains underwent isotropic relaxation, reorganizing into their original configurations. This structural reorganization highlighted the critical role of chain mobility and alignment in driving the shape memory behavior of PLA/PEG blends, enabling them to effectively return to their initial shape. Mechanical testing confirmed that increasing PEG content and molecular weight enhanced elongation at break and impact strength, balancing flexibility and strength. These findings demonstrated that PLA/PEG blends, especially with 15 wt% PEG at 12,000 g/mol, offer an optimal combination of shape memory performance and mechanical properties, positioning them as promising candidates for customizable and biodegradable medical applications.

## 1. Introduction

Shape memory polymers (SMPs) are a remarkable class of smart materials with the ability to “remember” and revert to their original shape after deformation when triggered by external stimuli such as temperature, water, light, etc. [1,2,3,4,5,6,7,8,9,10]. SMPs have vast potential applications, from self-deploying spacecraft components and smart textiles to medical devices and self-disassembling electronics [11,12]. Structurally, SMPs typically comprise two key components: a switching phase and a rigid phase. The rigid phase stores energy during deformation and defines the permanent shape, while the switching phase consists of molecular switches responsive to external stimuli. Upon deformation into a temporary shape, SMPs develop additional transient network points within the polymer matrix, which facilitate the maintenance of this temporary form until an external stimulus is applied [10,13].

Among various types of SMPs, thermoresponsive SMPs are particularly noteworthy. These polymers can be controlled using thermomechanical forces when heated. They can be programmed to temporarily form different shapes while in a rubbery state [14]. The process typically involves transforming the polymer from its original shape at a temperature higher than the transition temperature (T_trans_) and subsequently cooling it to a temperature below T_trans_ to achieve the temporary form. Reheating the polymer above the T_trans_ threshold causes it to undergo shape recovery and return to its initial state. The T_trans_ refers to either the T_g_ or the melting temperature (T_m_) of the polymer. Notably, some SMPs can also be programmed at room temperature, depending on the material properties and specific applications.

Poly(lactic acid) (PLA), a thermoplastic polymer synthesized from lactic acid monomers, has gained significant attention in the field of SMPs. PLA possesses exceptional mechanical properties, biocompatibility, and biodegradability. Its shape memory behavior arises from a delicate interplay between crystalline and amorphous phases. The crystalline phase serves as the fixed points responsible for storing energy during the temporary shape formation, while the amorphous phase initiates shape switching above the T_g_ [15,16]. Although PLA has numerous advantages, its inherent brittleness restricts its use in applications requiring high flexibility and toughness. Moreover, the relatively high T_g_ (around 60 °C) poses a significant constraint for its utilization as a shape memory polymer, particularly in the field of biomedical applications. This is due to the risk of tissue damage when heat is applied at 44 °C or above [17].

To overcome the mentioned drawbacks of PLA, several strategies have been proposed to improve the performance of PLA-based SMPs. These include copolymerization [18,19], surface modification [20,21,22], and blending [10,13,23,24]. Among these, blending plasticizers with PLA is considered more economical and environmentally friendly. Plasticizers are employed to enhance the processability, flexibility, and ductility of PLA. This is accomplished by allowing the plasticizers to penetrate between the PLA chains, thereby enhancing chain mobility and improving its elongation at break and T_g_.

Several plasticizers were used to prepare the PLA/plasticizer blend, such as tributyl citrate (TBC) [25,26,27], acetyl tributyl citrate (ATBC) [28,29,30], and oligomeric lactic acid (OLA) [31,32,33]. Among these, poly(ethylene glycol) (PEG), a non-toxic and biodegradable plasticizer, has gained particular interest. Previous research has shown that PEG can significantly modify PLA’s mechanical properties and maintain the shape memory behavior [13,34,35]. For instance, Li et al. investigated the effects of PEG content (0–20 wt%) and molecular weight (6000–20,000 g/mol) in PLA/PEG blends, finding that higher-molecular-weight PEG increased tensile modulus and strength while reducing elongation at break of the blends [34]. The blend with 10 wt% PEG at 10,000 g/mol showed optimal miscibility, chain mobility, and toughness. Similarly, Guo et al. studied a PLA/PEG blend with various PEG molecular weights (0.6–20 kg/mol) and contents (10–20 wt%), reporting that elongation at break of the blend increased with PEG content up to 15 wt%, with minimal impact from molecular weight [35]. However, the PLA/PEG blend with 15 wt% PEG at 5000 g/mol exhibited significantly lower elongation at break (32%) compared to ~300% for the blend with other molecular weights with the same blend content. WAXD and DSC analyses showed that lower-molecular-weight PEG improved the miscibility of the PLA/PEG blend, while higher molecular weights suppressed phase separation through entanglement. Additionally, Guo et al. explored the shape memory behavior of PLA/PEG blend with various PEG content. They found that adding 5 wt% PEG reduced T_g_ from 59.5 °C (neat PLA) to 50 °C and increased elongation at break from 18% to 800%, with excellent shape memory properties retained [13]. These findings highlighted the potential of PLA/PEG blends for biomedical applications, as they demonstrated improved elongation at break, thermal properties, and shape memory performance.

While significant progress has been made in biodegradable SMPs, research has predominantly focused on external parameters such as draw ratio, tensile strain, programming temperature (T_p_), and recovery temperature (T_r_). The influence of plasticizer content and molecular weight on the recovery behavior of PLA/PEG blends, particularly the associated microstructural changes, remains underexplored. Investigating the microstructural evolution during deformation and recovery is essential to understanding mechanisms like phase interactions and crystalline–amorphous transitions, which drive the thermally stimulated shape memory effect. These insights are key to optimizing SMP performance and expanding their biomedical applications.

This study aimed to investigate the effects of PEG content and molecular weight on the shape memory behavior of PLA/PEG blends, focusing on the thermal, microstructural, and mechanical mechanisms driving this behavior. Shape memory tests were conducted at a programming temperature (T_p_) of 45 °C and a recovery temperature (T_r_) of 60 °C, conditions chosen for their relevance to human skin applications and their ability to effectively activate shape memory properties. Thermal properties, including T_g_ and crystallinity, were analyzed using differential scanning calorimetry (DSC) to elucidate PEG’s role in enhancing chain mobility, driving structural reorganization, and facilitating shape fixity and recovery. To explore the microstructural dynamics of PLA/PEG blends, synchrotron wide-angle X-ray scattering (WAXS) was employed to examine real-time structural changes across three key states: original, post-stretching, and recovery. WAXS provided detailed insights into chain alignment during stretching and isotropic relaxation during recovery, highlighting PEG’s influence on modulating crystalline and amorphous regions. Additionally, mechanical properties of the PLA/PEG blends were tested to identify the formulation that optimized strength and flexibility while preserving effective shape memory performance. These analyses highlighted the potential of PLA/PEG blends for biomedical applications, offering novel insights into optimizing their properties.

## 2. Materials and Methods

### 2.1. Materials

Poly(*L*-lactic acid) (PLA), under trade name Ingeo™ Biopolymer 4043D with *D*-isomer content = 4.2%, was purchased from Nature Works LLC (Minneapolis, MN, USA). Poly(ethylene glycol) (PEG) with average molecular weights of 4000, 8000, and 12,000 g/mol was purchased from Thermo Scientific (Waltham, MA, USA).

### 2.2. Preparation of Neat PLA and PLA/PEG Blends

The neat PLA and PLA/PEG blends with various PEG contents (5–20 wt%) and molecular weights (M_W_) (4000–12,000 g/mol) were prepared using a twin-screw extruder with a die diameter of 3 mm (Charoen Tut Model CTE-D16L32, Samutprakarn, Thailand) (L/D = 32). The mixing temperature from die to feed zone was 175, 175, 180, 185, 175, 170, 165, 155, and 50 °C. The screw speed was 50 rpm. The extrudate was cut into small pellets using a pelletizer. All test specimens were produced by compression molding (Charoen Tut Model: PR1D-W300L350-PM-WCL-HMI, Samutprakarn, Thailand) at 170 °C for 7 min under a pressure of 125 MPa.

### 2.3. Characterization of Neat PLA and PLA/PEG Blends

#### 2.3.1. Shape Memory Behavior

Shape memory properties of the PLA/PEG blends were evaluated by assessing two parameters: shape fixity ratio (R_f_) and shape recovery ratio (R_r_). The R_f_ quantifies the ability of the specimen to maintain temporary deformations induced during the programming step. The R_r_ quantifies the extent to which the specimens revert to their original shape, indicating their capacity for shape recovery.

**Figure 1:**
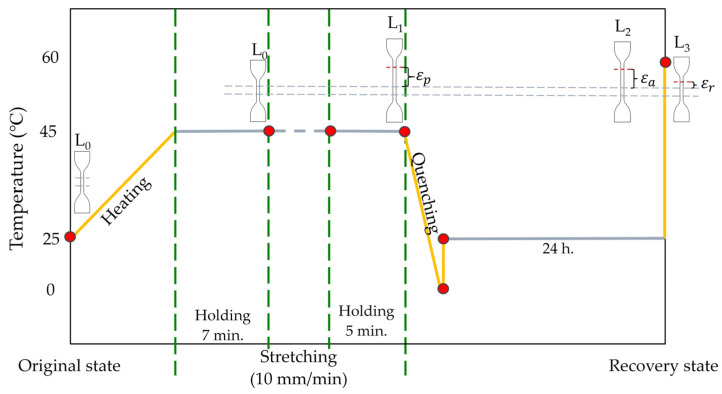
Shape memory test procedure showing temperature profile and specimen deformation stages. L_0_ to L_3_ indicate sample lengths at different states, used to derive the corresponding strains.

Shape memory tests were conducted using a universal testing machine (UTM) (Instron 5569 Model, Norwood, MA, USA) equipped with a heated chamber. Figure 1 depicts the stage of the shape memory test. The following procedures were carried out: (1) The specimen, which had an initial length of L_0_, was subjected to a constant T_p_ of 45 °C for a duration of 7 min. (2) The specimen was subsequently stretched to a strain of 100% at a rate of 10 mm/min. The length of the specimen, L_1_, was recorded to calculate the strain prior to unloading, E_p_. The stretched specimen was held at a temperature of 45 °C for a duration of 5 min. Following that, the specimen was rapidly cooled using an ice pack. (3) The specimen was removed and stored at an ambient temperature for 24 h. The length of the specimen after aging, L_2_, was used to determine the strain after aging, E_a_. (4) The stretched specimens were submerged in a water bath at a T_r_ of 60 °C to assess the specimen’s recoverability. The length of the specimen after recovery, L_3_, was used to calculate the residual strain after recovery, denoted as E_r_. Experiments were performed on a minimum of five specimens for each experimental condition. The R_f_ and R_r_ values of the specimens were calculated according to the method described in reference [36].

(1) $$R_{f}=\left(\frac{E_{a}}{E_{p}}\right)\times 100$$

(2) $$R_{r}=\left(\frac{E_{a}-E_{r}}{E_{a}}\right)\times 100$$

Here E_p_, E_a_, and E_r_ represent the level of the strain before unloading, after aging, and residual strain after recovery, respectively.

#### 2.3.2. Thermal Characteristics

Thermal properties of the specimens, both before and after stretching as well as after recovery, were determined using a differential scanning calorimeter (DSC, DSC 3 + STARe System, Mettler Toledo, Schwerzenbach, Switzerland). The observed parameters included T_g_, T_m_, cold crystallization temperature (T_cc_), and degree of crystallinity (X_c_). Small sections of specimens, representing their original, stretched, and recovered states, were subjected to a single heating step from 25 to 200 °C at a rate of 10 °C/min under a nitrogen atmosphere. The X_c_ of each sample was estimated using the following Equation (3):

(3) $$X_{c}\left(\%\right)=\frac{∆H_{m}-∆H_{cc}}{\omega ∆H_{m}^{o}}\times 100$$

where ω is the weight fraction of PLA, and ∆H_m_ and ∆H_cc_ are the enthalpies of melting and cold crystallization, respectively. ∆$H_{m}^{o}$ is melting enthalpy of 100% crystalline PLA (93.7 J/g) [10].

#### 2.3.3. Microstructure Evaluation

Synchrotron WAXS measurement was conducted on beamline 1.3W from Synchrotron Light Research Institute (Public Organization), located in Nakhon Ratchasima, Thailand, to study the microstructural evolution of specimens during a shape memory test. This study focused on analyzing the specimens at three specific states: original, stretched, and recovered. X-ray energy of 9 keV (λ = 1.38 Å) was applied, with an exposure duration of 60 s. The specimen-to-detector distance for WAXS measurements was 138.38 mm. The WAXS data were processed using the SAXSIT Version 4.63 software. The crystallinity index (X_c-WAXS_) of the drawn specimens was calculated using the following Equation (4) [19]:

(4) $$X_{c-WAXS}\left(\%\right)=\frac{A_{c}}{A_{c}+A_{a}}\times 100$$

where A_a_ and A_c_ are integral intensities of amorphous halos and crystalline peaks, respectively.

In addition, an azimuthal scan from the peak intensity of the PLA (200)/(110) planes was employed to monitor polymer chain alignment during shape memory experiments. The Hermans orientation factor (*f*_c_), calculated from the azimuthal intensity in scattering experiments, was used to quantify the alignment level of polymer chains, as described in previous studies [37]. Here, an *f*_c_ value close to 0 indicates a randomly oriented or isotropic structure, while an *f*_c_ value of 1 denotes perfect uniaxial orientation [37].

#### 2.3.4. Mechanical Properties

Tensile properties of neat PLA and PLA/PEG blends were determined using a universal testing machine (UTM) (Instron 5569 Model, Norwood, MA, USA). The UTM was operated at room temperature and utilized a 5 kN load cell. Testing was performed with a crosshead speed set to 10 mm/min, consistent with the procedures outlined for Type V specimens in ASTM D638 [38]. For each experimental condition, a minimum of five replicates were examined to obtain the average values.

The impact strength of neat PLA and PLA/PEG blends was determined using a notch-Izod impact tester (Instron, Wycombe, UK) with a 2.75 J hammer, in accordance with ASTM D256 [39]. At least five replicates of specimens from each experimental condition were tested to obtain the average values.

#### 2.3.5. Morphology

The morphologies of neat PLA and PEG/PLA blends were examined using a scanning electron microscope (SEM) (JEOL JSM-6010LV, Peabody, MA, USA) at a voltage of 5 kV. The impact-fractured specimens were coated with gold for 3 min to ensure suitable electrical conductivity.

## 3. Results and Discussion

### 3.1. Shape Memory Behavior of Neat PLA and PLA/PEG Blends

**Figure 2:**
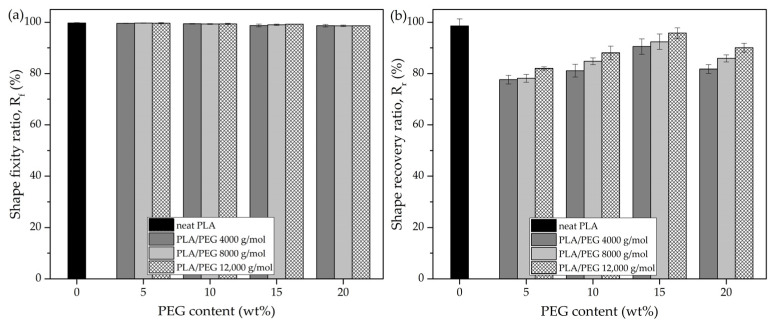
(**a**) Shape fixity ratio (R_f_) and (**b**) shape recovery ratio (R_r_) of neat PLA and PLA/PEG blends as a function of PEG content (0–20 wt%) and molecular weight (0–12,000 g/mol).

Shape memory properties of neat PLA and PLA/PEG blends, evaluated through the shape fixity ratio (R_f_) and shape recovery ratio (R_r_), are presented in Figure 2. The results, shown in Figure 2a, revealed that the R_f_ of neat PLA (≈99%) was comparable to that of PLA/PEG blends, with no significant changes observed across varying PEG contents and molecular weights. This suggested that heating and stretching facilitated polymer chain alignment and crystallinity. The application of heat before stretching enhanced the mobility of chain segments, while the stretching process aligned the polymer chains along the direction of tension. This alignment reduced chain entanglement, increased crystallinity, and decreased spacing and disorder in the amorphous regions [40]. These structural changes contributed to the consistent ability of the blends to retain temporary shapes.

In contrast, Figure 2b presents the R_r_, where neat PLA exhibited the highest value (98.6%). This was likely due to its high amorphous content, which allowed the polymer chains to more easily revert to a random coil configuration upon heating [40]. For the PLA/PEG blends, R_r_ improved with increasing PEG content, peaking at 15 wt% PEG (95.77%) before declining at higher contents. Similarly, blends with higher-molecular-weight PEG showed improved recovery, with the highest R_r_ observed for the PEG with a molecular weight of 12,000 g/mol. Notably, the blend containing 15 wt% PEG and a molecular weight of 12,000 g/mol demonstrated the best recovery performance among the PLA/PEG blends.

To further explore the mechanisms behind the superior R_r_ of the PLA/PEG blend with 15 wt% PEG (12,000 g/mol), DSC and synchrotron-WAXS analyses were conducted. These analyses focused on two variables: the effect of varying PEG content in blends with a fixed molecular weight of 12,000 g/mol and the effect of different PEG molecular weights with a constant PEG content of 15 wt%.

### 3.2. Thermal Properties of Neat PLA and PLA/PEG Blends

**Figure 3:**
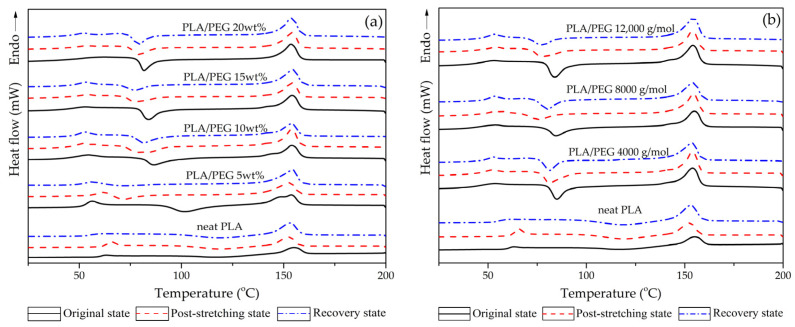
DSC thermograms of neat PLA and PLA/PEG blends at the original, post-stretching, and recovery states, showing (**a**) different PEG content (5–20 wt%) with a constant molecular weight of 12,000 g/mol and (**b**) different PEG molecular weights (4000, 8000, and 12,000 g/mol) with a fixed PEG content of 15 wt%.

DSC thermograms of neat PLA and PLA/PEG blends, prepared in three different states—original, post-stretching, and recovery—are illustrated in Figure 3. Figure 3a demonstrates the effects of varying PEG content (5–20 wt%) while maintaining a constant molecular weight of 12,000 g/mol. Conversely, Figure 3b shows the impact of different PEG molecular weights (4000–12,000 g/mol) on the blends with a constant 15 wt% content. The results of this analysis, detailing the thermal characteristics, i.e., glass transition temperature (T_g_), cold crystallization temperature (T_cc_), melting temperature (T_m_), and degree of crystallinity (X_c_), are summarized in Table 1.

In the original state, neat PLA exhibited T_g_, T_cc_, and T_m_ at 55.5 °C, 121.8 °C, and 152.0 °C, respectively. The addition of PEG of various contents and molecular weights significantly influenced these parameters. As PEG content increased, the T_g_ and T_cc_ of the PLA/PEG blends exhibited a significant decline while the X_c_ increased, with the blend containing 20 wt% PEG (12,000 g/mol) displaying the lowest T_g_ and T_cc_ but the highest X_c_ (17.3%). This trend was attributed to PEG’s ability to penetrate the amorphous phase of PLA, reducing the energy barrier for chain mobility and enabling more efficient chain rearrangement into crystalline structures [41,42,43]. With a fixed PEG content of 15 wt%, T_g_ and T_cc_ of the PLA/PEG blends slightly increased, while their X_c_ decreased with increasing PEG molecular weight. The longer PEG chains may have enhanced PLA–PEG interactions, contributing to the increased T_g_. However, the steric hindrance introduced by longer PEG chains may have limited the PLA chain rearrangement into a crystalline structure, reducing overall crystallinity [35,41,42,43].

In the post-stretching state, both the T_g_ and the X_c_ of neat PLA and PLA/PEG blends increased compared to their original state. While the programming temperature (45 °C) was lower than the T_g_ of neat PLA, holding the specimens at this temperature for 7 min allowed the polymer chains to become more flexible. This increased flexibility facilitated the orderly alignment of chains along the tensile force during stretching, enabling tighter packing of PLA/PEG chains. Consequently, the increase in T_g_ and X_c_ reflected the enhanced restriction of polymer chain mobility and improved fixation of the deformed shape. The higher crystallinity achieved after stretching contributed to a more stable temporary shape, enhancing the R_f_ [10,16,40,44].

During the recovery state, the specimens were stimulated at 60 °C, exceeding the T_g_ of PLA/PEG blends. For neat PLA, the T_g_ returned to 55.5 °C, matching its original state. This behavior occurred as the PLA chains relaxed and reoriented near T_g_, reverting to their amorphous structure. In PLA/PEG blends, increasing the PEG content reduced the T_g_ of the recovered specimens, resulting in a higher R_r_. The elevated recovery temperature improved chain mobility within the PLA matrix, particularly for blends with higher PEG content, enabling easier recovery. Moreover, X_c_ of both neat PLA and PLA/PEG blends increased during recovery compared to the post-stretching state. The increase in X_c_ was likely due to polymer chain relaxation and reorganization during immersion in hot water at 60 °C, which promoted chain rearrangement and a higher degree of crystallinity [40,44]. However, in the blends with low-molecular-weight PEG (4000 and 8000 g/mol), the T_g_ of the recovered specimens increased compared to their post-stretching state, resulting in a lower R_r_. In contrast, the blend with high-molecular-weight PEG (12,000 g/mol) exhibited lower T_g_ than in its post-stretched state. This allowed it to recover more effectively and achieve a higher R_r_. The observed T_g_ variations in the recovery state can be attributed to microstructural changes. Interestingly, higher PEG molecular weights were associated with lower X_c_, indicating a greater proportion of amorphous regions, which likely contributed to the higher R_r_ observed in PLA/PEG blends [13,44].

Despite the observed changes in T_g_ and X_c_ during the shape memory process, no significant variations were found in the T_m_. This suggested that the shape memory behavior in the PLA/PEG blends primarily involved changes in the amorphous phase and crystallinity, without affecting the overall melting characteristics of the materials.

The DSC results demonstrated the critical role of PEG content and molecular weight in influencing the thermal transitions and crystallinity of PLA/PEG blends in relation to their shape memory behavior.

### 3.3. Microstructure of Neat PLA and PLA/PEG Blends During Shape Memory Test

To gain a deeper understanding of the microstructural evolution during stretching and recovery, further investigation was conducted using synchrotron WAXS. This technique provided a detailed perspective on the chain orientation and the crystalline–amorphous dynamics that drive the shape memory behavior of both neat PLA and PLA/PEG blends. The specimens used in this analysis included neat PLA and PLA/PEG blends with varying PEG contents (5–20 wt%) and a fixed PEG molecular weight of 12,000 g/mol. Additionally, blends with different PEG molecular weights were examined at a fixed PEG content of 15 wt%. These formulations were selected based on earlier findings showing their influence on shape memory behavior, specifically R_r_.

**Figure 4:**
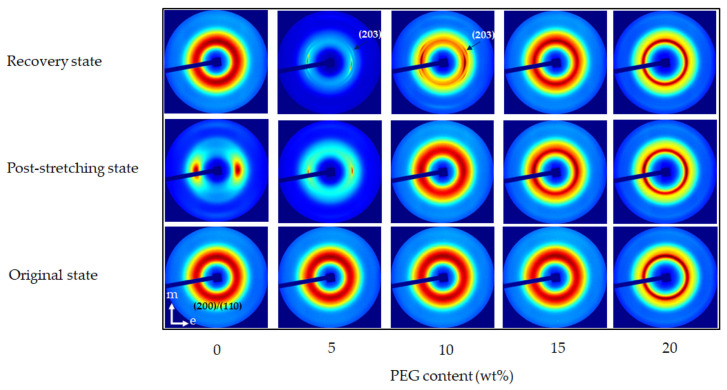
The 2D WAXS patterns of neat PLA and PLA/PEG blends at various PEG contents (5–20 wt%) with a constant PEG molecular weight of 12,000 g/mol during the shape memory test process.

**Figure 5:**
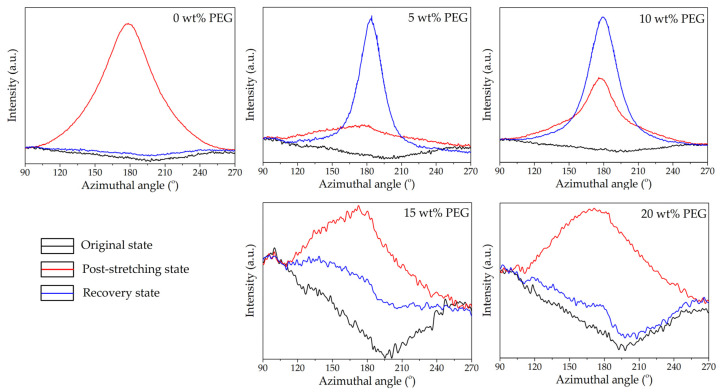
Azimuthal patterns of neat PLA and PLA/PEG blends at various PEG contents (5–20 wt%) with a constant PEG molecular weight of 12,000 g/mol during the shape memory test process.

**Figure 6:**
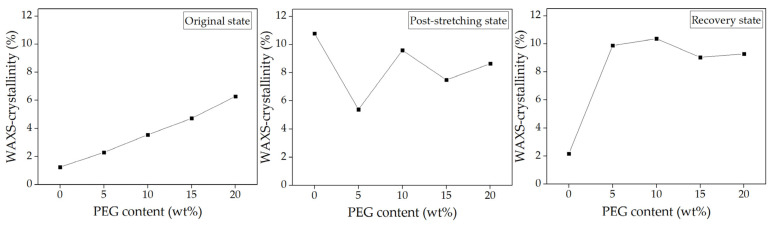
X_c-WAXS_ of neat PLA and PLA/PEG blends at various PEG contents (5–20 wt%) with a constant PEG molecular weight of 12,000 g/mol during the shape memory test process.

The analysis spanned three key states, i.e., original, post-stretching, and recovery, providing insights into how the material’s crystalline and amorphous regions responded to mechanical deformation and thermal stimuli. Two-dimensional (2D) WAXS patterns (Figure 4) provided a comprehensive perspective on chain alignment focusing on two principal axes: the meridian axis (m), aligned with the stretching force, and the equatorial axis (e), which was perpendicular to the force. The directional analysis elucidated the extent of chain orientation and deformation at different stages of the shape memory test. Azimuthal scans of the peak intensity from the PLA (200)/(110) planes were performed to monitor polymer chain alignment, with the results illustrated in Figure 5. Then, the Hermans orientation factor (*f*_c_), summarized in Table 2, was calculated from azimuthal intensity data to quantify polymer chain alignment. One-dimensional (1D) WAXS patterns were utilized to determine the crystallinity index (X_c-WAXS_), as shown in Figure 6, facilitating quantitative study of the crystalline phase.

#### 3.3.1. Microstructure of Neat PLA and PLA/PEG Blends with Varying PEG Contents

In the original state (Figure 4), both neat PLA and PLA/PEG blends displayed a diffuse amorphous halo, with slightly higher intensity along the meridional direction. This suggested a random arrangement of polymer chains before deformation. Azimuthal scans confirmed this, with maximum intensities between 92° and 95° along the meridional axis (Figure 5). The *f_c_* values were uniformly low across all specimens (Table 2), indicating predominantly random chain orientation. The X_c-WAXS_ in Figure 6 reveals that neat PLA had a lower crystallinity compared to the PLA/PEG blends. The addition of PEG increased crystallinity as PEG enhanced chain mobility and promoted crystallization [35,45]. These findings established the baseline microstructural properties of neat PLA and PLA/PEG blends before deformation.

After stretching at 45 °C, the 2D WAXS patterns of all specimens showed a clear shift toward anisotropy, with intensified scattering along the equatorial axis, as illustrated in Figure 4. Neat PLA exhibited distinct arcs along this axis, indicating significant PLA chain orientation due to mechanical stretching [19,37,46,47]. Moreover, the programming temperature, being below the T_g_ of PLA, prevented crystal formation but promoted the development of ordered mesocrystals or oriented molecular chains [47,48].

Following this, the azimuthal profiles of the post-stretching specimens in Figure 5 exhibited heightened intensities relative to their original states, with peak values ranging from 172° to 176° along the equatorial axis. Notably, neat PLA demonstrated the greatest *f*_c_ value of 0.146 (Table 2), suggesting a highly organized amorphous structure. In contrast, the *f*_c_ values for PLA/PEG blends decreased with increasing PEG content, reflecting increased chain flexibility at higher PEG contents (15–20 wt%).

From Figure 6, the post-stretching neat PLA specimen demonstrated the highest X_c-WAXS_ (10.79%), indicating its superior strain-induced alignment relative to the blends. The variations in X_c-WAXS_ across the PLA/PEG blends suggested the competing effects of PEG’s plasticizing and nucleating behaviors. At lower PEG contents (5–10 wt%), PEG facilitated chain mobility, enhancing crystallization. Conversely, at higher PEG contents (15–20 wt%), excessive chain mobility interfered with PLA chain packing, reducing crystallization. Consequently, the increased flexibility at elevated PEG contents led to more randomly oriented structures compared to blends with lower PEG content.

After recovery in hot water at 60 °C, the 2D WAXS patterns, shown in Figure 4, revealed significant microstructural differences between neat PLA and PLA/PEG blends. The diffraction pattern of neat PLA displayed an amorphous halo, closely resembling its original state. This demonstrated that the polymer chains had completely relaxed into a disordered configuration and relinquished any orientation acquired during stretching. In contrast, PLA/PEG blends with 5–10 wt% PEG retained a degree of anisotropy even after recovery. The anisotropic diffuse halo observed in the stretching state transformed into a discrete diffraction ring with small lobes along the equatorial direction. This suggested that the PEG phase facilitated the crystallization of PLA chains upon recovery at 60 °C, derived from the oriented structure formed during stretching. The presence of discrete equatorial diffractions of the (203) plane and small lobes of the (200)/(110) planes along the meridian further indicated partial disruption and breakdown of crystalline orientation. This reflected signs of overdrawing and structural damage from excessive stretching at low temperatures [49]. For PLA/PEG blends with higher PEG content (15–20 wt%), the 2D WAXS patterns after recovery showed a more isotropic structure compared to blends with lower PEG content. Interestingly, these blends showed no discrete structure of the (203) plane, suggesting that the stretching process did not cause structural damage. This improvement was attributed to the plasticizing effect of PEG, which reduced the T_g_ of the PLA component (Table 1), facilitating increased chain mobility during stretching at 45 °C.

The azimuthal profiles (Figure 5) and the *f*_c_ of the recovered specimens (Table 2) showed that the recovered PLA had low azimuthal intensity and a minimal *f*_c_ value (0.0042), which can be correlated with its highest recovery ratio (R_r_) of 98.6% (Figure 2). These results indicated that the PLA chains had relaxed completely into a disordered state, allowing the stored elastic energy to drive recovery to its original shape. In contrast, PLA/PEG blends with lower PEG content (5–10 wt%) exhibited relatively high azimuthal intensity and *f*_c_ values after recovery, suggesting that some polymer chains remained oriented and did not fully relax. However, for blends with higher PEG content (15–20 wt%), the azimuthal intensity and *f*_c_ values were similar to those in their original state, indicating superior recovery efficiency compared to those with lower PEG content.

The X_c-WAXS_ values of the recovered specimens (Figure 6) further substantiate these findings. The crystallinity of neat PLA dropped to 2.71%, closely matching its original state. This reduction aligned with the relaxation of PLA chains observed in the 2D WAXS and azimuthal profiles. Although neat PLA exhibited the highest T_g_ of 55.5 °C as compared to its blends, the initial crystallinity of PLA component in the blends was greater, as shown in Table 1. Our previous research demonstrated that PLA exhibited a strain-induced orientation effect rather than strain-induced crystallization when stretched at 45 °C [37]. Therefore, the polymer chains in the neat PLA specimen exhibited increased flexibility and mobility, leading to enhanced recoverability. PLA/PEG blends with low PEG content (5–10 wt%) demonstrated enhanced crystallinity compared to their post-stretching state; however, their T_g_ remained relatively high, limiting chain mobility during recovery. The existing crystalline domains underwent permanent deformation during stretching, resulting in irreversible changes that diminished their shape recovery ability. Incorporating increased PEG content, maximized at 15 wt% PEG, resulted in a reduction in the T_g_ of the PLA blends, facilitating the polymer chains’ ability to recover more readily and return to their initial configuration, which was associated with the higher R_r_. However, with the further addition of PEG up to 20 wt%, increased molecular relaxation may have occurred during the stretching process due to its T_g_ being near the stretching temperature. This led to permanent deformation, compromising the network points essential for shape recovery.

The incorporation of PEG of various contents in PLA blends significantly influenced the microstructural evolution across different stages of the shape memory process. Lower PEG content enhanced crystallinity but may have limited recovery due to insufficient chain mobility. In contrast, higher PEG contents facilitated better recovery and structural integrity, though very high contents might have led to reduced crystallinity due to excessive chain mobility. Optimal PEG content (around 15 wt%) appeared to strike a balance, providing sufficient plasticization to enhance recovery while maintaining structural coherence necessary for effective shape memory functionality.

#### 3.3.2. Microstructure of Neat PLA and PLA/PEG Blends with Varying PEG Molecular Weights

**Figure 7:**
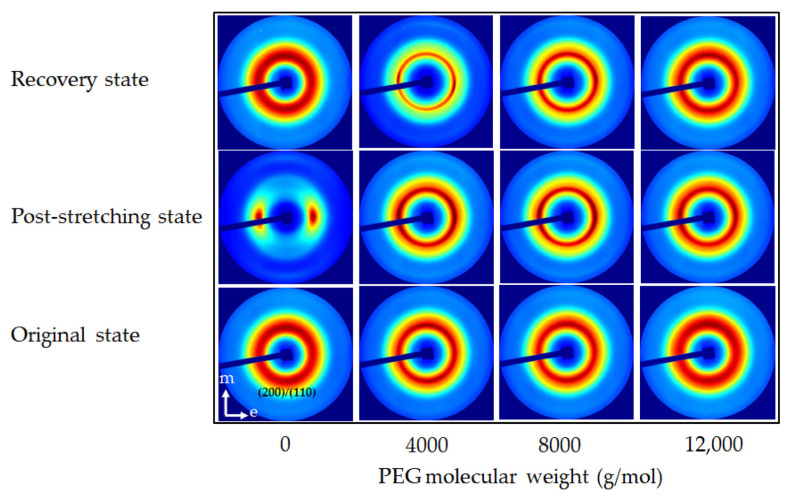
The 2D WAXS patterns of neat PLA and PLA/PEG blends at various PEG molecular weights with a constant PEG content of 15 wt% during shape memory test process.

WAXS was also utilized to examine the microstructural evolution of PLA/PEG blends with different PEG molecular weights, with neat PLA replotted for comparison. In their original state, the 2D WAXS of all specimens in Figure 7 showed a slightly oriented structure, as evidenced by the diffuse amorphous halo in the meridional direction. The peak azimuthal angle for PLA/PEG blends ranged between 93° and 95° in the meridional direction, as shown in Table 2. Additionally, as PEG molecular weight increased, the X_c-WAXS_ of PLA/PEG blends decreased. This was likely due to the increased chain entanglement between PLA and PEG, which restricted PLA chain mobility. This reduction in mobility led to lower crystallinity and, consequently, a decrease in X_c-WAXS_ for blends with higher PEG molecular weights [35,50].

**Figure 8:**
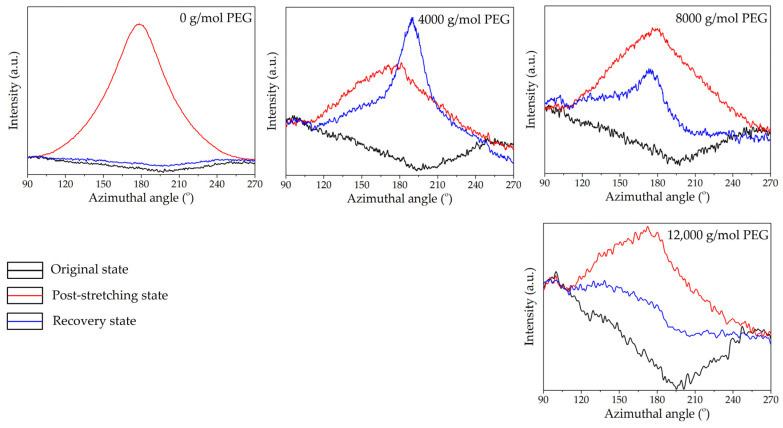
Azimuthal patterns of neat PLA and PLA/PEG blends at various PEG molecular weights with a constant PEG content of 15 wt% during shape memory test process.

**Figure 9:**
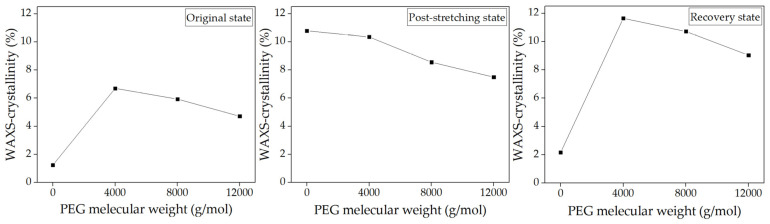
X_c-WAXS_ of neat PLA and PLA/PEG blends at various PEG molecular weights with a constant PEG content of 15 wt% during shape memory test process.

At the post-stretching state, the 2D WAXS patterns of PLA/PEG blends in Figure 7 showed an anisotropic arrangement with intense equatorial axis intensity, indicating significant chain alignment. This alignment was confirmed by the increased azimuthal intensity observed in their post-stretching state, as shown in Figure 8. The azimuthal angles reached approximately 172°–177° in the equatorial direction, as detailed in Table 2. The anisotropic alignment was less pronounced in PLA/PEG blends with higher PEG molecular weights. The incorporation of low-molecular-weight PEG decreased the T_g_ of the PLA blends relative to the high-molecular-weight PEG (Table 1); however, the low-molecular-weight PEG in these blends also led to an increase in X_c-WAXS_ (Figure 9). The increase in crystalline structure and the reduction in the amorphous phase produced a higher-intensity distribution, leading to a high *f*_c_ value. Therefore, as the molecular weight of PEG increased, the *f*_c_ value decreased.

High crystalline domains also appeared to impact the shape recovery behavior of PLA/PEG blends negatively. In the recovery state, the 2D WAXS patterns shown in Figure 7 illustrated that PLA/PEG blends with PEG molecular weights of 4000 and 8000 g/mol retained two arcs of oriented chains along the equatorial direction. This suggested that they maintained an anisotropic arrangement after recovery. The strong arcs were attributed to the PEG phase, which promoted PLA crystallization at 60 °C from the oriented structure induced during stretching. Consequently, the T_g_ of PLA/PEG blends with PEG molecular weights of 4000 and 8000 g/mol after recovery (Table 1) were higher than that of their post-stretched specimens.

The azimuthal intensity and *f*_c_ value, as shown in Figure 8 and Table 2, respectively, remained high for PLA blends with low-molecular-weight PEG, particularly at 4000 g/mol. In contrast, the PLA/PEG blend with 12,000 g/mol PEG exhibited an isotropic arrangement after recovery, with a low *f*_c_ value close to its original state, while the X_c-WAXS_ (Figure 9) remained the same. This indicated that the amorphous chains had fully relaxed and rearranged to their disordered configuration. This finding ascribed the rationale behind the superior R_r_ of the blend containing 12,000 g/mol PEG, surpassing that of blends with lower molecular weights (4000 and 8000 g/mol).

The shape recovery results (Figure 2b) were strongly supported by WAXS analysis, which confirmed that the PLA/PEG blend with 15 wt% PEG and a molecular weight of 12,000 g/mol exhibited the best recovery behavior. The WAXS data revealed superior chain reorientation and isotropic relaxation during recovery, which contributed to the high shape recovery ability observed in this blend.

### 3.4. Mechanical Properties of Neat PLA and PLA/PEG Blends

Tensile and impact properties of neat PLA and PLA/PEG blends were further examined to determine their applicability in practical scenarios. The selected formulations included PLA/PEG blends with PEG contents ranging from 5 to 20 wt% at a constant PEG molecular weight of 12,000 g/mol, and blends with varying molecular weights at a constant PEG content of 15 wt%. These formulations were selected based on earlier findings demonstrating their influence on shape memory behavior, particularly R_r_.

#### 3.4.1. Tensile Properties of Neat PLA and PLA/PEG Blends

**Figure 10:**
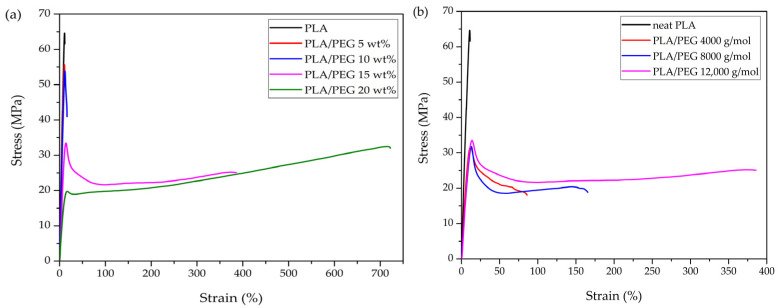
Stress–strain curves of neat PLA and PLA/PEG blends: (**a**) different PEG contents (5–20 wt%) with a constant molecular weight of 12,000 g/mol, and (**b**) different PEG molecular weights (4000, 8000, and 12,000 g/mol) with a fixed PEG content of 15 wt%.

Figure 10 shows tensile stress–strain curves of neat PLA and PLA/PEG blends as a function of PEG content and molecular weight. Their key tensile properties are summarized in Table 3. The neat PLA (0 wt% PEG) exhibited brittle failure with higher tensile strength (61.28 MPa) and tensile modulus (0.62 GPa) compared to PLA/PEG blends. However, it exhibited a lower level of elongation at break, at 12.79%.

Figure 10a illustrates the stress–strain curves of the PLA/PEG blends when maintaining a constant molecular weight at 12,000 g/mol. The PLA/PEG blends exhibited brittle failure at lower PEG contents (5–10 wt%). When the PEG content exceeded 10 wt%, the failure mode transitioned from brittle to ductile. The elongation at break of PLA/PEG blends demonstrated a positive correlation with increasing PEG content. This effect was particularly pronounced at higher PEG contents (15–20 wt%), where the blends exhibited markedly increased elongation compared to neat PLA. In contrast, at lower PEG contents (5–10 wt%), the impact was negligible.

At a constant PEG content, PLA/PEG blends of various PEG molecular weights exhibited ductile failure, as shown in Figure 10b. The elongation at break of PLA/PEG blends showed a direct correlation to the molecular weight of PEG. The addition of PEG with a higher molecular weight resulted in a substantial increase in the elongation at break of PLA/PEG blends. Within the 5–20 wt% range tested, the PLA/PEG blend achieved a maximum elongation at break of 740% with 20 wt% PEG at a molecular weight of 12,000 g/mol. This result aligned with the observed decrease in T_g_ in the original state of the specimens as PEG content increased. This decrease indicated enhanced chain flexibility in the PLA/PEG blends due to the addition of PEG, leading to improved elongation at break.

The tensile stress at yield and tensile modulus of PLA/PEG blends declined with increasing PEG content, with a significant drop observed when PEG content exceeded 15 wt%. The effect of molecular weight was also evident, with lower-molecular-weight PEG leading to more pronounced decreases in tensile properties. The blend containing 20 wt% PEG at 12,000 g/mol exhibited the lowest yield strength (23.87 MPa) and tensile modulus (0.22 GPa).

The significant increase in elongation at break and corresponding decrease in tensile strength and modulus at 15–20 wt% PEG content demonstrated PEG’s effectiveness as a plasticizer. PEG molecules penetrate between PLA chains, increasing their mobility [34,50]. Higher-molecular-weight PEG further improved chain flexibility, potentially due to the increased PLA–PEG interactions. This resulted in enhanced elongation at break and resistance to deformation in PLA/PEG blends [35], albeit at the cost of reduced tensile strength and modulus.

#### 3.4.2. Impact Strength of Neat PLA and PLA/PEG Blends

**Figure 11:**
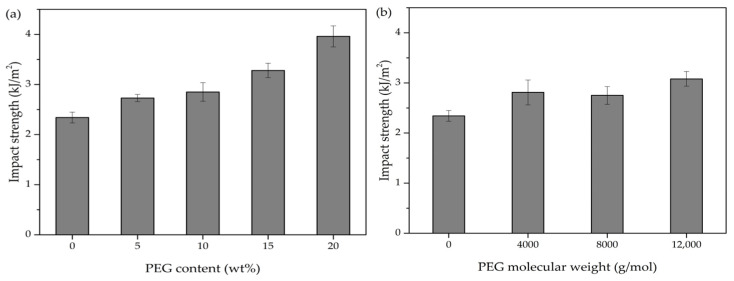
Impact strength of neat PLA and PLA/PEG blends: (**a**) different PEG contents (5–20 wt%) with a constant molecular weight of 12,000 g/mol, and (**b**) different PEG molecular weights (4000, 8000, and 12,000 g/mol) with a fixed PEG content of 15 wt%.

Figure 11 presents the impact strength of neat PLA and PLA/PEG blends. Neat PLA exhibited the lowest impact strength (2.34 kJ/m^2^), while the PLA/PEG blends demonstrated a clear improvement. The impact strength increased with increasing PEG content and PEG molecular weight, with each factor showing a positive correlation when the other was held constant. This increase in impact strength suggested enhanced energy absorption and distribution mechanisms within the polymer matrix. Among the PLA/PEG blends, the highest impact strength (3.96 kJ/m^2^) was observed in the PLA/PEG blend containing 20 wt% of PEG with a molecular weight of 12,000 g/mol.

The enhanced impact strength with increasing PEG content aligned with the observations from tensile testing, further confirming PEG’s role as a plasticizer. Moreover, the impact strength of PLA/PEG blends increased with higher-molecular-weight PEG. This implied that longer PEG chains led to increased entanglement in PLA/PEG blends [45]. These longer chains can aid in the dispersion of energy during impact occurrences, thereby enhancing the durability and impact resistance of the polymer blend.

### 3.5. Morphologies of Neat PLA and PLA/PEG Blends

**Figure 12:**
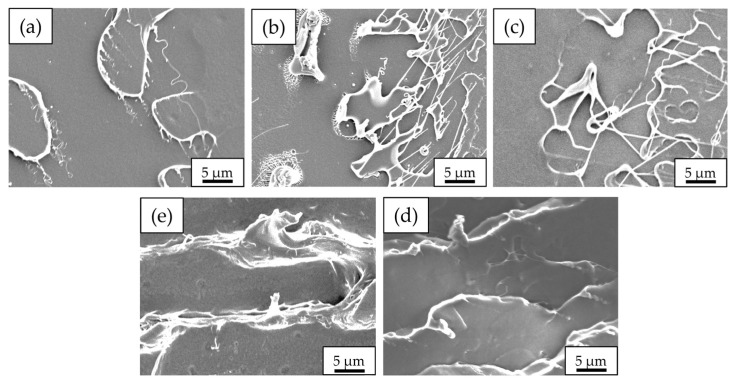
SEM micrographs of impact fracture surfaces (3000× magnification) of neat PLA and PLA/PEG blends as a function of PEG content with PEG molecular weight fixed at 12,000 g/mol: (**a**) neat PLA; PLA/PEG at (**b**) 5 wt%, (**c**) 10 wt%, (**d**) 15 wt%, and (**e**) 20 wt%.

**Figure 13:**
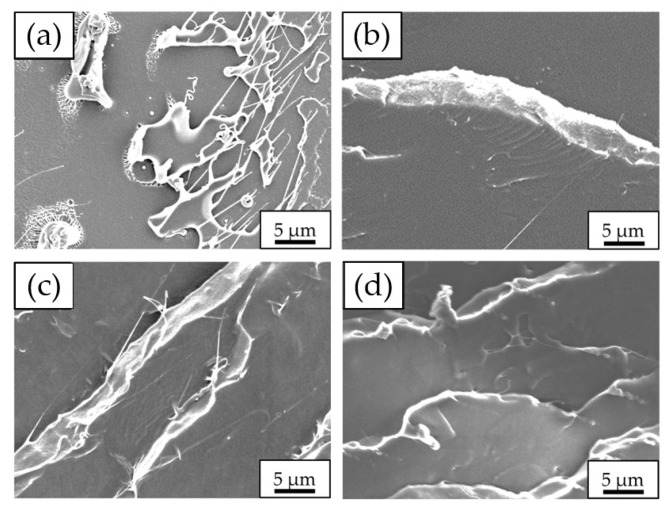
SEM micrographs of impact fracture surfaces (3000× magnification) of neat PLA and PLA/PEG blends as a function of PEG molecular weight with PEG content fixed at 15 wt%: (**a**) neat PLA; PLA/PEG at (**b**) PEG 4000 g/mol, (**c**) PEG 8000 g/mol, and (**d**) PEG 12,000 g/mol.

SEM micrographs of the impact fracture surface of neat PLA and PLA/PEG blends at various PEG contents and molecular weights are shown in Figure 12 and Figure 13, respectively. The SEM micrograph in Figure 12a shows the impact fracture surface of neat PLA, which was rather smooth, with a few short fibrils. This indicated the brittle nature of the specimen. This observation was consistent with the neat PLA’s low impact strength and low elongation at break, as discussed in earlier sections.

The fracture surfaces of PLA/PEG blends displayed a higher number of fibrils and roughness compared to that of neat PLA. PLA/PEG blends with 5 to 10 wt% PEG content (Figure 12b,c) exhibited smooth and uniform fracture surfaces, indicating a characteristic of brittle failure. In contrast, increasing the PEG content to 15–20 wt% (Figure 12d,e) resulted in noticeably rougher surfaces, signifying a shift from brittle to ductile failure behavior. This morphological change correlated with the significant improvements in elongation at break and impact strength observed at higher PEG contents.

Additionally, the SEM micrographs of PLA/PEG blends revealed higher fibril content and increased roughness as the PEG molecular weight increased, as presented in Figure 13b–d). This observation aligns with the enhanced impact strength seen in the blends with higher-molecular-weight PEG, as discussed in earlier sections. The increased roughness and fibril formation can be attributed to the enhanced mobility of PLA chains due to the addition of PEG. When subjected to impact loads, these more mobile PLA chains can slip more easily, leading to increased plastic deformation and energy dissipation. Furthermore, the inclusion of high-molecular-weight PEG resulted in increased chain entanglement between PLA and PEG chains [45], contributing to the improved mechanical properties.

The combined effects of PEG content and molecular weight on both tensile properties and impact strength highlighted the versatility of PEG in modifying PLA’s characteristics. PEG’s role as a plasticizer is critical in enhancing flexibility, elongation at break, and impact resistance of PLA, while simultaneously reducing tensile strength and modulus. The observed trends in mechanical property improvements and morphological changes aligned with findings reported by Li et al. [34], Shin et al. [50], and Gao et al. [51] in various polymer blending systems.

## 4. Conclusions

This study highlighted the significant impact of PEG content and molecular weight on shape memory properties, microstructural behavior, and mechanical properties of PLA/PEG blends. This provided novel insights into optimizing the blend for potential use in biomedical applications. Shape memory tests revealed that the blend with 15 wt% PEG and a molecular weight of 12,000 g/mol exhibited the highest shape recovery ratio (R_r_) of 95.77%, outperforming other PLA/PEG blends. Thermal analysis further supported these results, showing that increasing PEG content reduced T_g_ and increased PLA crystallinity, facilitating shape memory performance. However, the higher-molecular-weight PEG in the PLA/PEG blend caused steric hindrance. This resulted in a slight reduction in crystallinity, while simultaneously promoting superior chain flexibility and recovery. Synchrotron WAXS analysis revealed PLA/PEG chain alignment during stretching and isotropic relaxation during recovery, emphasizing the interplay between crystallinity and amorphous content. These findings demonstrated that higher-molecular-weight PEG enhanced chain mobility and structural reorganization, improving the shape recovery of the PLA/PEG blend. Mechanical testing confirmed that blends with higher PEG content, particularly 12,000 g/mol PEG at 15 wt%, exhibited improved elongation and impact strength while transitioning from brittle to ductile failure modes, as observed in SEM analysis. Despite neat PLA’s high shape recovery (98.6%), its brittleness and low flexibility limit its applications. The PLA/PEG blend with 15 wt% PEG and 12,000 g/mol achieved the best combination of shape memory properties, microstructural stability, and mechanical performance, making it a promising candidate for biomedical applications such as thermoresponsive medical splints. Future work will explore composite designs to further enhance its mechanical properties and expand its potential for advanced biomedical applications.

## Figures and Tables

**Table 1:** Key thermal characteristics of neat PLA and PLA/PEG blends at the original, post-stretching, and recovery states.

PEG MW (g/mol)	PEG Content (wt%)	Original State			Post-Stretching State			Recovery State		
		T_g_ (°C)	T_m_ (°C)	X_c_ (%)	T_g_ (°C)	T_m_ (°C)	X_c_ (%)	T_g_ (°C)	T_m_ (°C)	X_c_ (%)
0	0	55.5	152.0	1.7	62.5	152.3	6.0	55.5	153.0	7.4
12,000	5	55.2	153.7	5.1	58.8	152.8	15.2	53.3	154.5	16.3
12,000	10	53.0	153.8	11.1	51.3	153.8	17.4	50.8	153.8	21.2
12,000	15	51.3	154.2	14.2	51.0	153.8	21.6	50.5	154.3	25.7
12,000	20	48.3	153.5	17.3	49.7	153.7	24.1	49.7	153.5	27.4
4000	15	49.3	153.8	15.2	49.7	153.8	23.4	50.8	153.7	27.4
8000	15	48.5	154.8	14.3	49.6	154.3	23.4	51.2	154.0	26.7
12,000	15	51.3	154.2	14.2	51.0	153.8	21.6	50.5	154.3	25.7

**Table 2:** Maximum azimuthal angle and Hermans orientation factor of neat PLA and PLA/PEG blends at the original state, post-stretching, and recovery states.

PEG MW (g/mol)	PEG Content (wt%)	Maximum Azimuthal Angle (°)			Hermans Orientation Factor, *f*_c_		
		Original State	Post-Stretching State	Recovery State	Original State	Post-Stretching State	Recovery State
0	0	93.2	176.4	95.1	0.0059	0.1463	0.0042
12,000	5	94.7	176.1	179.4	0.0061	0.0366	0.0155
12,000	10	94.3	176.7	173.7	0.0062	0.0108	0.0183
12,000	15	95.5	172.6	88.6	0.0066	0.0072	0.0068
12,000	20	92.5	172.6	95.4	0.0068	0.0072	0.0070
4000	15	95.4	177.1	189.6	0.0072	0.0156	0.0147
8000	15	93.9	176.6	179.6	0.0069	0.0114	0.0096
12,000	15	95.5	172.6	88.6	0.0066	0.0072	0.0068

**Table 3:** Key tensile properties of neat PLA and PLA/PEG blends.

PEG MW (g/mol)	PEG Content (wt%)	Tensile Stress at Yield (MPa)	Modulus (GPa)	Elongation at Break (%)
0	0	61.28 ± 3.52	0.62 ± 0.07	12.79 ± 1.05
12,000	5	59.31 ± 1.02	0.61 ± 0.02	13.29 ± 1.17
12,000	10	55.44 ± 0.66	0.55 ± 0.05	16.72 ± 2.26
12,000	15	35.26 ± 0.06	0.36 ± 0.04	484.48 ± 5.54
12,000	20	23.87 ± 1.42	0.22 ± 0.02	740.88 ± 19.69
4000	15	33.77 ± 0.92	0.31 ± 0.02	69.74 ± 4.39
8000	15	34.14 ± 0.10	0.34 ± 0.03	164.44 ± 6.51
12,000	15	35.26 ± 0.06	0.36 ± 0.04	484.48 ± 5.54

## Data Availability

Data are contained within the article.

## References

[1] Fulati A., Uto K., Ebara M. (2022). Influences of Crystallinity and Crosslinking Density on the Shape Recovery Force in Poly(ε-Caprolactone)-Based Shape-Memory Polymer Blends. Polymers.

[2] Staszczak M., Nabavian Kalat M., Golasiński K.M., Urbański L., Takeda K., Matsui R., Pieczyska E.A. (2022). Characterization of Polyurethane Shape Memory Polymer and Determination of Shape Fixity and Shape Recovery in Subsequent Thermomechanical Cycles. Polymers.

[3] Bai Y., Chen X. (2017). A fast water-induced shape memory polymer based on hydroxyethyl cellulose/graphene oxide composites. Compos. Part A Appl. Sci. Manuf..

[4] Meng D., Zhao Q., Cheng X., Ma J., Kong L., He X., Li J. (2022). Water-induced shape memory cellulose nanofiber-based nanocomposite membrane containing lignin with quick water response and excellent wet mechanical property. Eur. Polym. J..

[5] Lendlein A., Jiang H., Jünger O., Langer R. (2005). Light-induced shape-memory polymers. Nature.

[6] Li M.-H., Keller P., Li B., Wang X., Brunet M. (2003). Light-Driven Side-On Nematic Elastomer Actuators. Adv. Mater..

[7] Wang Y., Chen Z., Niu J., Shi Y., Zhao J., Ye J., Tian W. (2020). Electrically Responsive Shape Memory Composites Using Silver Plated Chopped Carbon Fiber. Front. Chem..

[8] Huang X., Panahi-Sarmad M., Dong K., Li R., Chen T., Xiao X. (2021). Tracing evolutions in electro-activated shape memory polymer composites with 4D printing strategies: A systematic review. Compos. Part A Appl. Sci. Manuf..

[9] van Vilsteren S.J.M., Yarmand H., Ghodrat S. (2021). Review of Magnetic Shape Memory Polymers and Magnetic Soft Materials. Magnetochemistry.

[10] Nonkrathok W., Trongsatitkul T., Suppakarn N. (2022). Role of Maleic Anhydride-Grafted Poly(lactic acid) in Improving Shape Memory Properties of Thermoresponsive Poly(ethylene glycol) and Poly(lactic acid) Blends. Polymers.

[11] Suman T., Leng J. (2017). Shape Memory Polymers for Smart Textile Applications. Textiles for Advanced Applications.

[12] Xu J., Song J., Fazel-Rezai R. (2011). Thermal Responsive Shape Memory Polymers for Biomedical Applications. Biomedical Engineering—Frontiers and Challenges.

[13] Guo Y., Ma J., Lv Z., Zhao N., Lixia W., Li Q. (2018). The effect of plasticizer on the shape memory properties of poly(lactide acid)/poly(ethylene glycol) blends. J. Mater. Res..

[14] Alam F., Ubaid J., Butt H., El-Atab N. (2023). Swift 4D printing of thermoresponsive shape-memory polymers using vat photopolymerization. NPG Asia Mater..

[15] Dogan S.K., Boyacioglu S., Kodal M., Gokce O., Ozkoc G. (2017). Thermally induced shape memory behavior, enzymatic degradation and biocompatibility of PLA/TPU blends: “Effects of compatibilization”. J. Mech. Behav. Biomed. Mater..

[16] Nie D., Yin X., Cai Z., Wang J. (2022). Effect of Crystallization on Shape Memory Effect of Poly(lactic Acid). Polymers.

[17] Verma A., Rath P., Mahapatra S. (2017). Assessment of Thermal Damage During Skin Tumor Treatment Using Thermal Wave Model: A Realistic Approach. J. Heat Transf..

[18] da Cunha R.B., Cavalcanti S.N., Agrawal P., de Figueiredo Brito G., de Mélo T.J.A. (2023). Evaluation of shape memory effect in PLA/copolymers blends. J. Appl. Polym. Sci..

[19] Koosomsuan W., Yamaguchi M., Phinyocheep P., Sirisinha K. (2019). High-Strain Shape Memory Behavior of PLA–PEG Multiblock Copolymers and Its Microstructural Origin. J. Polym. Sci. Part B Polym. Phys..

[20] Rasal R.M., Janorkar A.V., Hirt D.E. (2010). Poly(lactic acid) modifications. Prog. Polym. Sci..

[21] Govindarajan T., Shandas R. (2014). A Survey of Surface Modification Techniques for Next-Generation Shape Memory Polymer Stent Devices. Polymers.

[22] Zhang L., Lin Z., Zhou Q., Ma S., Liang Y., Zhang Z. (2020). PEEK modified PLA shape memory blends: Towards enhanced mechanical and deformation properties. Front. Mater. Sci..

[23] Ji X., Gao F., Geng Z., Li D. (2021). Fabrication of thermoplastic polyurethane/polylactide shape-memory blends with tunable optical and mechanical properties via a bilayer structure design. Polym. Test..

[24] Zhang S., Liu T., Zhao B., Verdi C., Liu W., Hao C., Zhang J. (2020). Shape memory Poly(lactic acid) binary blends with unusual fluorescence. Polymer.

[25] Hassan M., Mustapa I., Daud N., Nahida J.H., Sudin N. (2019). Effects of Tributyl Citrate Plasticizer on Thermomechanical Attributes of Poly Lactic Acid. J. Adv. Res. Fluid Mech. Therm. Sci..

[26] Chaos A., Sangroniz A., González A., Iriarte M., Sarasua J.-R., del Río J., Etxeberria A. (2018). Tributyl citrate as an effective plasticizer for biodegradable polymers: Effect of plasticizer on free volume and transport and mechanical properties. Polym. Int..

[27] Lemmouchi Y., Murariu M., dos Santos A., Amass A., Schacht E., Dubois P. (2009). Plasticization of poly(lactide) with blends of tributyl citrate and low molecular weight poly(D,L-lactide)-b-poly(ethylene glycol) copolymers. Eur. Polym. J..

[28] Aliotta L., Canesi I., Lazzeri A. (2021). Study on the preferential distribution of acetyl tributyl citrate in poly(lactic) acid-poly(butylene adipate-co-terephthalate) blends. Polym. Test..

[29] He R., Tao Y., Luo Z., Yang L., Liao J., Xu M., Yang S., Lin Y. (2024). Properties of polylactic acid/gelatin/acetyl tributyl citrate blend materials. J. Appl. Polym. Sci..

[30] Stoll L., Domenek S., Hickmann Flôres S., Nachtigall S.M.B., de Oliveira Rios A. (2021). Polylactide films produced with bixin and acetyl tributyl citrate: Functional properties for active packaging. J. Appl. Polym. Sci..

[31] Avolio R., Castaldo R., Gentile G., Ambrogi V., Fiori S., Avella M., Cocca M., Errico M. (2015). Plasticization of poly(lactic acid) through blending with oligomers of lactic acid: Effect of the physical aging on properties. Eur. Polym. J..

[32] Gomez-Caturla J., Tejada-Oliveros R., Ivorra J., Garcia-Sanoguera D., Balart R., Garcia D. (2024). Development and Characterization of New Environmentally Friendly Polylactide Formulations with Terpenoid-Based Plasticizers with Improved Ductility. J. Polym. Environ..

[33] Burgos N., Tolaguera D., Fiori S., Jiménez A. (2014). Synthesis and Characterization of Lactic Acid Oligomers: Evaluation of Performance as Poly(Lactic Acid) Plasticizers. J. Polym. Environ..

[34] Li F.-J., Liang J.-Z., Zhang S.-D., Zhu B. (2015). Tensile Properties of Polylactide/Poly(ethylene glycol) Blends. J. Polym. Environ..

[35] Guo J., Liu X., Liu M., Han M., Liu Y., Ji S. (2021). Effect of molecular weight of Poly(ethylene glycol) on plasticization of Poly(l-lactic acid). Polymer.

[36] Luengrojanakul P., Mora P., Bunyanuwat K., Jubsilp C., Rimdusit S. (2023). Improvements in Mechanical and Shape-Memory Properties of Bio-Based Composite: Effects of Adding Carbon Fiber and Graphene Nanoparticles. Polymers.

[37] Kajornprai T., Sringam J., Seejuntuek A., Kaewsuwan D., Kamonsutthipaijit N., Chio-Srichan S., Suppakarn N., Trongsatitkul T. (2025). Crystal Evolution of Amorphous Poly(lactic acid) During Simultaneous Multi-step Tensile Deformation and Annealing. J. Polym. Sci..

[38] (2014). Standard Test Method for Tensile Properties of Plastics.

[39] (2010). Standard Test Methods for Determining the Izod Pendulum Impact Resistance of Plastics.

[40] Wei X., Wang Z., Tian Z., Luo T. (2021). Thermal Transport in Polymers: A Review. J. Heat Transf..

[41] Sungsanit K., Kao N., Bhattacharya S.N. (2012). Properties of linear poly(lactic acid)/polyethylene glycol blends. Polym. Eng. Sci..

[42] Athanasoulia I.-G., Tarantili P. (2016). Preparation and characterization of polyethylene glycol/poly(L-lactic acid) blends. Pure Appl. Chem..

[43] Kumar R., Alex Y., Nayak B., Mohanty S. (2023). Effect of poly (ethylene glycol) on 3D printed PLA/PEG blend: A study of physical, mechanical characterization and printability assessment. J. Mech. Behav. Biomed. Mater..

[44] Wong Y.S., Venkatraman S.S. (2010). Recovery as a measure of oriented crystalline structure in poly(l-lactide) used as shape memory polymer. Acta Mater..

[45] Li F.-J., Zhang S.-D., Liang J.-Z., Wang J.-Z. (2015). Effect of polyethylene glycol on the crystallization and impact properties of polylactide-based blends. Polym. Adv. Technol..

[46] Jariyasakoolroj P., Tashiro K., Wang H., Yamamoto H., Chinsirikul W., Kerddonfag N., Chirachanchai S. (2015). Isotropically small crystalline lamellae induced by high biaxial-stretching rate as a key microstructure for super-tough polylactide film. Polymer.

[47] Perret E., Hufenus R. (2021). Insights into strain-induced solid mesophases in melt-spun polymer fibers. Polymer.

[48] Zhou C., Li H., Zhang W., Li J., Huang S., Meng Y., Christiansen J.d., Yu D., Wu Z., Jiang S. (2016). Direct investigations on strain-induced cold crystallization behavior and structure evolutions in amorphous poly(lactic acid) with SAXS and WAXS measurements. Polymer.

[49] Heeley E., Billimoria K., Parsons N., Figiel Ł., Keating E., Cafolla C., Crabb E., Hughes D. (2020). In-situ uniaxial drawing of poly-L-lactic acid (PLLA): Following the crystalline morphology development using time-resolved SAXS/WAXS. Polymer.

[50] Shin H., Thanakkasaranee S., Sadeghi K., Seo J. (2022). Preparation and characterization of ductile PLA/PEG blend films for eco-friendly flexible packaging application. Food Packag. Shelf Life.

[51] Gao C., Wang B., Hu Z., Liu Y., Wang H., Zhang X., Wu Y. (2018). Effect of the molecular weight on the plasticization properties of poly(hexane succinate) in poly(vinyl chloride) blends. J. Appl. Polym. Sci..

